# Neurobiology and medico-legal aspects of suicides among older adults: a narrative review

**DOI:** 10.3389/fpsyt.2024.1449526

**Published:** 2024-09-02

**Authors:** Francesco Sessa, Rita Polito, Giuseppe Li Rosi, Monica Salerno, Massimiliano Esposito, Daniela Pisanelli, Federica Ministeri, Antonietta Messina, Marco Carotenuto, Sergio Chieffi, Giovanni Messina, Marcellino Monda

**Affiliations:** ^1^ Department of Medical, Surgical and Advanced Technologies “G.F. Ingrassia”, University of Catania, Catania, Italy; ^2^ Department of Clinical and Experimental Medicine, University of Foggia, Foggia, Italy; ^3^ Faculty of Medicine and Surgery, “Kore” University of Enna, Enna, Italy; ^4^ Microbiology and Virology Unit, Ospedali Riuniti, Viale Luigi Pinto, Foggia, Italy; ^5^ Department of Precision Medicine, University of Campania, Luigi Vanvitelli, Napoli, Italy; ^6^ Clinic of Child and Adolescent Neuropsychiatry, Department of Mental Health, Physical and Preventive Medicine, University of Campania “Luigi Vanvitelli”, Naples, Italy; ^7^ Department of Clinical Medicine, University of Campania, Luigi Vanvitelli, Napoli, Italy

**Keywords:** aging, neurobiology, medico-legal aspects, suicides, older adult, populations

## Abstract

The task of preventing suicide in older adults is an important social burden as older adults aged above 65 are exposed to singular psychological aspects that increase suicide risks. Moreover, when an older adult corpse is found, the medico-legal inspection represents a fundamental tool to identify the exact cause of death, classifying or excluding it as suicide. In this scenario, this review aims to explore the neurobiological factors that could be related to suicidal behavior in older adults. A further goal of this review is the exploration of the medico-legal aspects surrounding older adult suicides, clarifying the importance of forensic investigation. Particularly, this review examines issues such as neurotransmitter imbalances, cognitive impairment, neuroinflammation, psychosocial factors related to geriatric suicide, and neurodegenerative diseases. Additionally, medico-legal aspects such as policy considerations, legal frameworks, mental health assessments, ethical implications and forensic investigation were explored. Considering the importance of this phenomenon, especially in western countries, a need has emerged for focused screening tools on suicidal behavior among older adults, in order to contain it. Therefore, this review makes an exhaustive appraisal of the literature giving insights into the delicate interplay between neurobiology as well as mental health in relation to older adult suicide within a medico-legal context. The comprehension of different aspects about this complex phenomenon is fundamental to propose new and more effective interventions, supporting tailored initiatives such as family support and improving healthcare, specifically towards vulnerable ageing societies to reduce older adult suicide risks.

## 1 Introduction

Aging encompasses two interchangeable definitions: age-related decline in biological functions and age-related increase in mortality. Suicide among older adults is a critical public health concern. Individuals aged above 65 face unique mental health challenges, including increased suicidal behavior risks (1). Globally, according to the World Health Organization (WHO), one person dies from suicide every 40 seconds, with the highest rates observed among those over 65 years (2). Persistent concerns involve social isolation, loneliness, and addiction as significant risk factors for suicide (3).

Epidemiologically, older adults, particularly those over 65, exhibit higher rates of completed suicide compared to other age groups (4, 5). Suicide rates among the older adults progressively increase with age, especially among men. In 2017, rates were 16.17 per 100,000 for ages 50–69 and 27.45 per 100,000 for ages 70 and older (1, 6). Men in this demographic group have notably higher completion rates, whereas women tend to attempt suicide more frequently (4, 5). Physical health challenges, chronic illnesses, social isolation, and experiences of bereavement contribute significantly to their vulnerability (7).

In developed countries, people aged 65–74 generally maintain good health and social inclusion, though by 2050, two-thirds of the global older adult population will reside in low- and middle-income countries (8). The COVID-19 pandemic raised concerns about its impact on geriatric suicides, potentially exacerbating anxiety, depression, and post-traumatic stress symptoms (9–11). Understanding neurobiological factors in geriatric suicide is crucial, including neurotransmitter imbalances such as serotonin and dopamine (DA) dysregulation linked to depressive states and suicidal ideation (12). Chronic neuroinflammation, exacerbated by systemic illnesses and aging, contributes to cognitive decline and emotional instability (13). These factors underscore the need for targeted interventions to mitigate older adult suicide risk.

When an older adult corpse is discovered, a medico-legal inspection becomes an essential tool in the investigation process. This thorough examination is vital for accurately determining the cause of death and plays a crucial role in classifying or excluding the possibility of suicide (14). The importance of such inspections cannot be overstated, as they provide a comprehensive understanding of the circumstances surrounding the death, ensuring that justice is served, and families receive accurate information. The presence of pre-existing medical conditions, medications, and age-related changes must be carefully considered to avoid misclassification of the cause of death (5). Furthermore, medico-legal inspections contribute to the broader understanding of patterns and trends in older adult deaths. By accurately classifying deaths as suicides or otherwise, forensic data can be used to inform public health strategies and preventive measures. This information is crucial for developing targeted interventions aimed at reducing suicide rates among older adults, a demographic often vulnerable to mental health issues and social isolation (15, 16).

The complexity of understanding suicide ideation and behavior in this population involves aging-related factors, neurobiology, and legal considerations. This review explores neurobiological contributors to geriatric suicide, focusing on neurotransmitter imbalances, neuroinflammation, cognitive impairment, and neurodegenerative diseases, alongside psychosocial factors. It also examines medico-legal aspects, including forensic investigations, legal frameworks, policy considerations, ethical implications, and mental health assessments.

## 2 Materials and methods

A comprehensive search was conducted across the electronic databases PubMed/MEDLINE, PsycINFO, Scopus, and Google Scholar. Key search terms included “geriatric suicide”, “neurobiological factors”, “neurotransmitter imbalance”, “neuroinflammation”, “cognitive impairment”, “neurodegenerative diseases”, “psychosocial factors”, “medico-legal aspects”, “forensic investigation”, “legal framework”, “policy considerations”, “ethical implications”, and “mental health assessments”. Articles and reviews published in English between 2000 and 2024 were included. Emphasis was placed on studies focusing on neurobiological mechanisms of suicidal behavior in older adults and medico-legal aspects of geriatric suicide.

### 2.1 Study selection

Titles and abstracts of retrieved articles were screened for relevance to the topic. Full texts of potentially relevant articles were then assessed for eligibility. Studies and reviews that provided substantial insights into neurobiological factors contributing to suicidal behavior in older adults, as well as those discussing medico-legal considerations, were included. Studies focusing on pediatric populations, non-human subjects, or lacking relevance to the review’s scope were excluded. This methodology facilitated a comprehensive exploration of neurobiological mechanisms and medico-legal considerations surrounding geriatric suicide, contributing to the understanding of factors influencing suicidal behavior in older adults.

## 3 Neurobiological factors contributing to suicidal behavior in older adults

**Figure 1:**
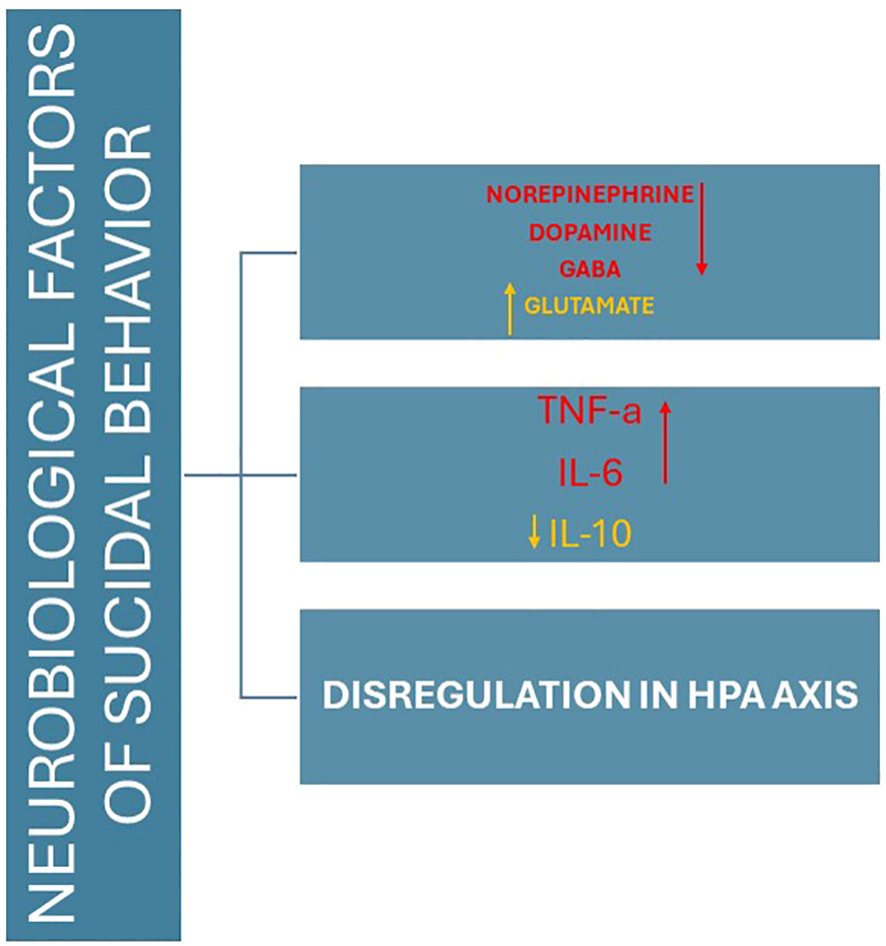
The main neurobiological factors involved in older adult suicides.

Suicidality, encompassing suicidal thoughts, behaviors, and completed suicide, is a severe public health concern and a leading cause of death worldwide. As reported in **Figure 1**, neurobiological factors play a pivotal role in suicidal behavior, particularly in older adults. Understanding the underlying mechanisms of suicidality is crucial for developing effective prevention and intervention strategies. Mental disorders, including major depressive disorder (MDD), bipolar disorder (BD), schizophrenia, and anxiety disorders, are significantly associated with increased risk of suicide. These disorders are characterized by complex neurobiological alterations that contribute to the emergence and progression of suicidal behavior (17–19). Research has identified several key neurobiological factors implicated in suicidality across various mental disorders. These include dysregulation of neurotransmitter systems, structural brain abnormalities, hypothalamic-pituitary-adrenal (HPA) axis dysfunction, genetic predispositions, and neuroinflammation. For instance, imbalances in serotonin, norepinephrine (NE), and DA levels have been consistently linked to mood disturbances and impulsivity, which are common precursors to suicidal acts. Additionally, structural changes in brain regions involved in emotion regulation and decision-making, such as the prefrontal cortex, amygdala, and hippocampus, have been observed in individuals with a history of suicide attempts (20, 21). The HPA axis, a central component of the body’s stress response system, is often dysregulated in those at risk for suicide, leading to abnormal cortisol levels and heightened stress reactivity. Genetic studies have also revealed specific polymorphisms that may predispose individuals to suicidality by affecting neurotransmitter function and inflammatory pathways (20). Moreover, chronic neuroinflammation, marked by elevated pro-inflammatory cytokines and microglial activation, has emerged as a significant factor contributing to the pathophysiology of suicidality (22).

By elucidating these complex mechanisms, we can better understand the etiology of suicidal behavior and pave the way for innovative treatment and prevention strategies tailored to the neurobiological profiles of at-risk individuals (20).

### 3.1 Neurotransmitter imbalance

Neurotransmitter imbalances play a critical role in suicidal behavior among older adults. These imbalances affect mood regulation, cognitive function, and stress responses, which are crucial in understanding the pathophysiology of suicidal behavior. In this context, drug assumption may negatively influence mood regulation systems (23–25). Alterations in neurotransmitter systems, particularly serotonin and NE, play a significant role in the pathophysiology of suicidal behavior (26). In the older adult population, age-related changes in these neurotransmitter systems may contribute to an increased vulnerability to suicidal ideation and actions: - serotonin (5-HT) is integral to mood regulation, anxiety, and impulse control (27). Platelet serotonin levels are commonly used as a peripheral marker of serotonergic function. Studies have shown reduced platelet serotonin levels in suicidal individuals compared to non-suicidal controls (28, 29). Lower levels of 5-HIAA (5-hydroxyindoleacetic acid), a primary serotonin metabolite, in the blood are also associated with increased suicidality. Reduced serotonergic activity is consistently linked to increased suicidal behavior. Studies have found lower levels of serotonin and its primary metabolite, 5-hydroxyindoleacetic acid (5-HIAA), in the cerebrospinal fluid of individuals who have attempted or completed suicide. Furthermore, low serotonin levels can impair impulse control and increase aggression, contributing to the risk of suicidal actions (24, 30). Moreover, several older adults could be homozygous for the “s” allele in the serotonin transporter promoter polymorphism (5HTTLPR): The 5HTTLPR gene, which is involved in serotonin transport, has been extensively studied in relation to how it may influence an individual’s response to life stressors. Variations in the 5HTTLPR gene affect serotonin reuptake, thereby influencing serotonin availability in the brain. Research suggests that individuals with certain variants of the 5HTTLPR gene may have different responses to stress compared to those with other variants. Specifically, the short allele (S allele) of the 5HTTLPR gene has been associated with increased susceptibility to stress-related disorders. Individuals carrying the S allele may exhibit heightened emotional reactivity and vulnerability to developing depression and anxiety disorders in response to stressful life events. On the other hand, carriers of the long allele (L allele) tend to show more resilience and adaptive responses to stressors (31).

The interplay between genetic variants of 5HTTLPR and environmental factors, such as life stressors, underscores the complex nature of how genes and environment interact to influence mental health outcomes, this polymorphism may negatively influence life stressors, predicting adverse mental health outcomes such as depressive symptoms (32, 33). In addition, cerebrospinal fluid (CSF) levels of 5-HIAA (5-hydroxy indole acetic acid) have been extensively studied, revealing lower concentrations in suicidal individuals. It was found that low CSF 5-HIAA levels were significantly associated with violent suicidal behavior, suggesting a central serotonergic deficit (32, 33). DA is another important neurotransmitter involved in reward processing, motivation, and pleasure (34). Dysregulation in the dopaminergic system can lead to anhedonia (loss of pleasure), decreased motivation, and depressive symptoms, which are prominent risk factors for suicide. Altered dopamine transmission can disrupt reward circuits, leading to feelings of hopelessness and reduced life satisfaction (35). Peripheral measures of DA activity, including plasma levels of homovanillic acid (HVA), a major DA metabolite, provide indirect insights into central dopaminergic function. Reduced plasma HVA levels have been associated with suicidal behavior in depressed patients (36). CSF studies have shown decreased levels of CSF HVA in suicidal individuals, particularly in those with major depressive disorder and schizophrenia. These findings suggest a deficit in central dopaminergic activity (35). NE is crucial for stress response, arousal, and mood regulation. Abnormal noradrenergic function is associated with mood disorders and suicidal behavior (37). Elevated or dysregulated NE levels can exacerbate anxiety and stress. Imbalances in NE can lead to hyperarousal, irritability, and heightened stress responses, increasing suicidal ideation (38). Altered levels of NE and its metabolites such as 3-methoxy-4-hydroxyphenylglycol (MHPG) in plasma have been reported in suicidal individuals. Other authors observed elevated plasma MHPG levels in suicidal patients with major depression, indicating heightened noradrenergic activity. CSF MHPG levels have also been studied as a marker of central noradrenergic function (39). Elevated CSF MHPG levels have been found in individuals with a history of suicide attempts suggesting dysregulation in NE turnover (38).

Glutamate is the primary excitatory neurotransmitter in the brain, involved in synaptic plasticity and cognitive function. Abnormal glutamatergic signaling has been linked to depression and suicidal behavior. Elevated glutamate levels can cause neurotoxicity and neuronal damage. Excessive glutamate activity can lead to excitotoxicity, impairing brain function and contributing to mood disturbances and cognitive deficits (38). Gamma-aminobutyric acid (GABA) is the main inhibitory neurotransmitter, crucial for reducing neuronal excitability and maintaining balance in brain activity. Reduced GABAergic activity has been associated with depression and anxiety, which are risk factors for suicide (40, 41). Low GABA levels can lead to increased neural excitability, anxiety, and stress, exacerbating suicidal tendencies (38).

Neurotransmitter imbalances play a critical role in the pathophysiology of suicidality. Studies of neurotransmitter metabolites in body fluids such as blood and CSF provide essential insights into central neurotransmitter function. Continued research in this area is crucial for developing effective strategies to identify, treat, and prevent suicidal behavior. The study of biomarkers in individuals with suicidal tendencies involves examining various body fluids and tissues to identify neurobiological alterations associated with suicidality.

### 3.2 Neuroinflammation

Chronic neuroinflammation has been linked to various neuropsychiatric disorders, including depression and anxiety, which are significant risk factors for suicide. In older adults, the presence of neuroinflammatory processes may exacerbate psychiatric symptoms, thereby increasing the risk of suicidal behavior (42, 43). Neuroinflammation plays a significant role in the pathophysiology of suicidal behavior, particularly in older adults. Chronic inflammation can influence brain function and mood regulation, leading to increased vulnerability to depression and suicidal tendencies (44, 45). Cytokine dysregulation plays an important role in neuroinflammation establishment (42). Elevated levels of pro-inflammatory cytokines such as interleukin-6 (IL-6), tumor necrosis factor-alpha (TNF-α), and interleukin-1 beta (IL-1β) are commonly observed in individuals with major depressive disorders and suicidal behavior (46, 47). These cytokines can cross the blood-brain barrier and affect brain regions involved in mood regulation, such as the prefrontal cortex and hippocampus, leading to altered neurotransmission and neuronal function (48). Neuroinflammation has been increasingly recognized as a significant factor in the pathophysiology of suicidality. Inflammatory markers in body fluids such as blood and CSF can provide valuable insights into the neurobiological mechanisms underlying suicidal behavior. Blood studies are commonly used to measure systemic inflammation and provide indirect insights into central nervous system (CNS) inflammation. Elevated levels of cytokines such as IL-6, IL-1β, and TNF-α have been found in the blood of suicidal individuals. Lindqvist et al. found significantly higher plasma levels of IL-6 and TNF-α in suicide attempters compared to non-suicidal depressed individuals and healthy controls (49). Janelidze et al. reported increased IL-1β and IL-6 levels in the blood of suicide attempters, suggesting a strong association between systemic inflammation and suicidality (50). Moreover, C-reactive protein (CRP) is a general marker of inflammation and has been associated with suicidality. Elder et al. found elevated CRP levels in individuals with suicidal ideation, indicating an association between systemic inflammation and suicidal thoughts (51). In addition, CSF studies provide direct insights into CNS inflammation and are critical for understanding the central mechanisms of suicidality. Increased levels of pro-inflammatory cytokines in CSF reflect neuroinflammation directly within the CNS. Lindqvist et al. reported elevated CSF levels of IL-6 in suicide attempters, indicating central inflammation (52). Moreover, Pandey et al. found increased levels of IL-1β and TNF-α in the CSF of suicide victims, further supporting the role of central neuroinflammation in suicidality (53). Neuroinflammation markers in body fluids, particularly elevated levels of pro-inflammatory cytokines and microglial activation markers, play a crucial role in the pathophysiology of suicidality. Studies of these markers in blood and CSF provide valuable insights into the systemic and central inflammatory processes associated with suicidal behavior (54).

Furthermore, microglial activation contributed to neuroinflammation. Microglia are the brain’s resident immune cells. In response to chronic stress or systemic inflammation, microglia can become overactivated, releasing pro-inflammatory cytokines and neurotoxic substances (55, 56). Persistent microglial activation can result in neuroinflammation, synaptic dysfunction, and neuronal damage, contributing to mood disturbances and cognitive impairments associated with suicidal behavior (48). In addition, chronic stress activates the hypothalamic-pituitary-adrenal (HPA) axis, leading to prolonged cortisol release. Elevated cortisol levels can exacerbate inflammatory responses. Dysregulation of the HPA axis and chronic inflammation can synergistically impair brain function and increase the risk of depression and suicidal behavior (57). There is evidence linking neuroinflammation and suicidal behavior, in fact, in postmortem analyses of individuals who died by suicide often reveal increased levels of pro-inflammatory cytokines and signs of microglial activation in brain tissues. These findings support the role of neuroinflammation in the pathophysiology of suicidal behavior (57).

Furthermore, neuroimaging studies using positron emission tomography (PET) scans have shown increased markers of neuroinflammation in the brains of individuals with suicidal ideation and behavior. These studies provide *in vivo* evidence of the link between neuroinflammation and suicidal tendencies (58–60). Regarding clinical studies, elevated inflammatory markers in blood samples such as C-reactive protein and IL-6 are often found in patients with depression and those with a history of suicide attempts (59). Systemic inflammation may reflect neuroinflammatory processes contributing to suicidal behavior. There are some important aspects such as age-related changes, in particular, aging is associated with increased systemic inflammation (often referred to as “inflammaging”), which can exacerbate neuroinflammatory processes (61, 62). In addition, older adults often have comorbid conditions such as cardiovascular diseases, diabetes, and neurodegenerative disorders, which can contribute to increased systemic and neuroinflammation (63). The combined effects of aging, comorbidities, and neuroinflammation can increase the vulnerability of older adults to depression and suicidal behavior. In this scenario, potential interventions are anti-inflammatory treatments such as non-steroidal anti-inflammatory drugs and cytokine inhibitors to reduce systemic and neuroinflammation (64). Moreover, lifestyle modifications promoting physical activity, a balanced diet, and stress management techniques are recommended to attenuate inflammation (65). Furthermore, some antidepressants have anti-inflammatory properties and can help reduce cytokine levels and neuroinflammation. Omega-3 fatty acids have been shown to have anti-inflammatory effects and can improve mood symptoms (66). Cognitive-behavioral therapy (CBT) and other psychotherapeutic interventions can help manage stress and reduce inflammatory responses (65). Over the last years the development of online and personal computer-based CBT programs may have been helpful in reducing suicides (67).

In summary, neuroinflammation is a critical factor in the development of suicidal behavior in older adults. Understanding the mechanisms and impacts of neuroinflammation can guide the development of targeted interventions to reduce the risk of suicide in this vulnerable population. Comprehensive approaches that combine pharmacological treatments, lifestyle modifications, and psychotherapeutic interventions are essential for effectively addressing neuroinflammation and its contribution to suicidal behavior (65, 68).

### 3.3 Cognitive impairment and neurodegenerative diseases

Older adults with dementia face unique challenges that may predispose them to suicidal tendencies. Cognitive impairment and neurodegenerative diseases significantly contribute to suicidal behavior in this age group (69). Particularly, cognitive decline, loss of autonomy, and the emotional burden on caregivers can increase the risks of suicidal ideation (70).

Structural changes in the brain associated with neurodegenerative diseases, such as Alzheimer’s disease (AD), have been implicated in the emergence of psychiatric symptoms and suicidal behavior in older adults (71, 72). These conditions affect cognitive function, emotional regulation, and overall quality of life, increasing the risk of depression and, as a consequence, suicidal ideation.

There are various forms of cognitive impairment. Mild cognitive impairment (MCI) represents a transitional stage between normal aging and dementia. It is characterized by noticeable cognitive decline that does not markedly interfere with daily functioning (73, 74). Dementia, on the other hand, entails severe cognitive decline affecting memory, thinking, and behavior to the extent that it disrupts daily activities (75).

In the early stages of cognitive decline, individuals are often aware of their diminishing cognitive abilities, leading to feelings of frustration, hopelessness, and depression, which can increase suicidal ideation. As cognitive impairment progresses, the ability to plan and execute complex actions, including suicide, may diminish. However, the distress and burden of living with cognitive decline can still contribute to suicidal thoughts (69, 76). In this context, AD represents the most common form of dementia, characterized by progressive memory loss, cognitive dysfunction, and behavioral changes (77, 78). Individuals with early-stage Alzheimer’s may experience depressive symptoms and suicidal ideation due to the awareness of their cognitive decline. Behavioral changes and reduced impulse control in later stages can also contribute to suicidal behavior. Moreover, Parkinson’s disease is a neurodegenerative disorder affecting motor function, often accompanied by cognitive impairment and mood disorders (79). Depression is common in Parkinson’s disease and significantly increases the risk of suicidal behavior. Cognitive impairment and reduced quality of life also contribute to this risk (80, 81). Frontotemporal dementia (FTD) involves the degeneration of the frontal and temporal lobes, leading to changes in personality, behavior, and language. Early onset and rapid progression of FTD can lead to severe emotional distress and impulsive behaviors, increasing the risk of suicidal actions (82). Lewy body dementia is characterized by the presence of Lewy bodies in the brain, leading to cognitive decline, visual hallucinations, and motor symptoms. The combination of cognitive impairment, hallucinations, and depression in Lewy body dementia can significantly increase the risk of suicidal behavior (83–85).

The perception of being a burden on caregivers can exacerbate feelings of worthlessness and hopelessness, contributing to suicidal ideation (86).

Neurodegeneration and dementia are complex conditions that often intersect with psychiatric disorders, including suicidality. Several studies demonstrated that patients diagnosed with dementia faced a higher risk of suicide within the first year compared to those without dementia (87, 88). For this reason, the identification of biomarkers in suicidal individuals with neurodegenerative diseases can provide insights into the underlying mechanisms and potentially guide interventions. Amyloid Beta (Aβ) Peptides are crucial in the pathology of AD and have been studied in relation to suicidality in neurodegenerative conditions. Reduced levels of Aβ42 in CSF are indicative of AD. Some studies suggest a correlation between low CSF Aβ42 and increased risk of suicidality. Other authors found that decreased CSF Aβ42 levels were associated with depressive symptoms and suicidal ideation in AD patients (89, 90). In addition, plasma levels of Aβ peptides are less reliable but may still provide useful information in conjunction with other biomarkers. Cai et al. showed that altered plasma Aβ levels, along with other markers, could predict cognitive decline and suicidal thoughts in older adults (91). Furthermore, Tau proteins, particularly total tau (t-tau) and phosphorylated tau (p-tau), are important markers for neurodegenerative diseases such as AD and FTD. Elevated CSF levels of t-tau and p-tau are associated with neuronal damage and have been linked to suicidality in dementia patients. Aamodt et al. reported higher CSF tau levels in patients with AD and FTD who exhibited suicidal behavior compared to those who did not (92). Advances in assay techniques have allowed for the detection of tau in plasma, providing a less invasive method to monitor neurodegeneration. Indeed, Mattsson et al. demonstrated that elevated plasma t-tau levels were correlated with increased risk of suicidal ideation in AD patients (93). Neurofilament Light Chain (NfL) is a marker of axonal damage and has been studied in various neurodegenerative conditions. Elevated CSF NfL levels are found in several neurodegenerative diseases and have been linked to the severity of neurodegeneration and suicidality. Soylu-Kucharz et al. found that increased CSF NfL levels were associated with both disease progression and suicidal tendencies in patients with neurodegenerative diseases (94). Blood levels of NfL are also elevated in neurodegenerative conditions and correlate with CSF levels, offering a non-invasive biomarker. Ashton et al. showed that plasma NfL levels could distinguish between suicidal and non-suicidal patients with neurodegenerative diseases (95).

Furthermore, synaptic dysfunction is a hallmark of neurodegeneration. Synaptic proteins serve as biomarkers for synaptic integrity and function. Proteins such as neurogranin and SNAP-25 are elevated in the CSF of patients with AD and other dementias and have been linked to suicidality. Kvartsberg et al. reported that increased CSF neurogranin levels were associated with cognitive decline and suicidal behavior in AD patients (96). Synaptic protein levels in blood are less commonly studied but may provide insights into synaptic health. Moreover, Ferrer-Cairols et al. found correlations between plasma neurogranin levels and suicidal ideation in dementia patients (97). In addition, as previously reported, neuroinflammation is significant in the progression of neurodegenerative diseases and has been linked to suicidality.

Biomarkers for neurodegeneration and dementia, detected in body fluids such as CSF and blood, provide critical insights into the interplay between neurodegenerative processes and suicidality. Key biomarkers include Aβ peptides, tau proteins, NfL, synaptic proteins, and inflammatory markers. Understanding these biomarkers’ roles can aid in diagnosing, monitoring, and potentially intervening in neurodegenerative diseases associated with suicidality.

### 3.4 Psychosocial factors

Older adults often face detachment from society and feel alone. These aspects are dangerous to the extent of bringing about depression as well as loneliness. In such cases, if an individual does not have any social networks, he or she may be overwhelmed by hopelessness and become so desperate that it can lead to suicidal thoughts and actions (98, 99).

Moreover, the mental health of older adults can be severely affected by chronic diseases, physical disabilities, and physical sicknesses, which, in turn, may raise the chances of suicidal behaviors (100). There are many psychosocial factors involved in this risk, which include a variety of social, psychological, and environmental influences that can impact mental health status and general well-being. Among older adults, feeling isolated and alone is a significant determinant of depression as well as being suicidal. The loss of a partner, friend, or family member can negatively influence their mental health status by causing a sense of extreme isolation or loneliness (100), increasing stress levels, decreasing mental health status, and deteriorating mental well-being. Different experiences, such as losing a loved one, especially a partner, are stressful events that often lead to the emergence of depressive disorders and suicidal thoughts. Sadness and loss make people feel that they are unfulfilled, unhappy, and have no reason to live, which hurts their psychological well-being. Chronic physical illness and the experience of persistent pain are well-documented and prevalent in older adults, who are highly vulnerable to depression and suicide (101). Such situations result in physical disability, substance abuse, and chronic pain, which eventually cause hopelessness and perceived burden. All these situations could be socially humiliating, making older adults unable to care for themselves: in this way, subjects increase their vulnerability, increasing depression and suicidal thoughts (102, 103). Financial stress and insecurity, often stemming from retirement, medical expenses, or loss of income, significantly impact mental health in older adults, generating anxiety, stress, and feelings of being a burden, increasing the risk of suicidal behavior (104). Depression, anxiety or previous suicide attempts are some of the mental health disorders that predict future suicidal behavior because mental health problems make other stress factors worse and decrease the ability to cope with change. Older adults could combine the consumption of alcohol and prescription medication, and this substance abuse increases the risk of suicide because it worsens depressive symptoms, affects judgment, and impulsive behavior (105, 106). The quality of relationships with family members and caregivers also influences mental health. Positive support can be protective, while conflict or perceived burden can increase suicide risk. Strained relationships and feelings of being a burden can led to worthlessness and hopelessness (107).

Daily living changes are challenging, and major life changes such as retirement, moving to a new place, or moving to a care home can be stressful and confusing. These transitions frequently lead to identity crises, role changes, and social isolation, which, in turn, give rise to depression and suicidal thoughts. Mental illness remains a taboo subject in many cultures, and there is still some shame involved in seeking help for a mental health issue (108, 109). Stigmatization by society will make such individuals develop feelings of shame and isolation, which will, in the long run, deny them any chance for help and thus increase their tendency towards suicide. In this case an effective approach to support and prevent is to promote the involvement in social activities and community programs to minimize isolation and loneliness (100). It should also be noted that the creation of opportunities for communication between individuals will also contribute to the significant improvement of the quality of life of older adults, including their sense of belonging and purpose. In addition, it should be made possible to get counseling and psychotherapy for grief, depression, and anxiety issues. Screening for depression and suicidal thinking should be carried out at regular intervals; early intervention is the best way to prevent dire consequences (110). It is also fundamental to ensure adequate medical treatment and pain management for chronic illnesses and offer physical therapy and rehabilitation services to improve functionality and quality of life. Managing physical health conditions effectively can reduce mental health strain (111, 112). Moreover, it would be helpful to provide guidance on financial matters to overcome challenges such as retirement, medical bills and financial pressure, as well as offering legal aid services to older adults who may be encountering issues with their finances or housing. Mental health problems can be eased by dealing with some financial problems to reduce the stressors associated with them.

**Figure 2:**
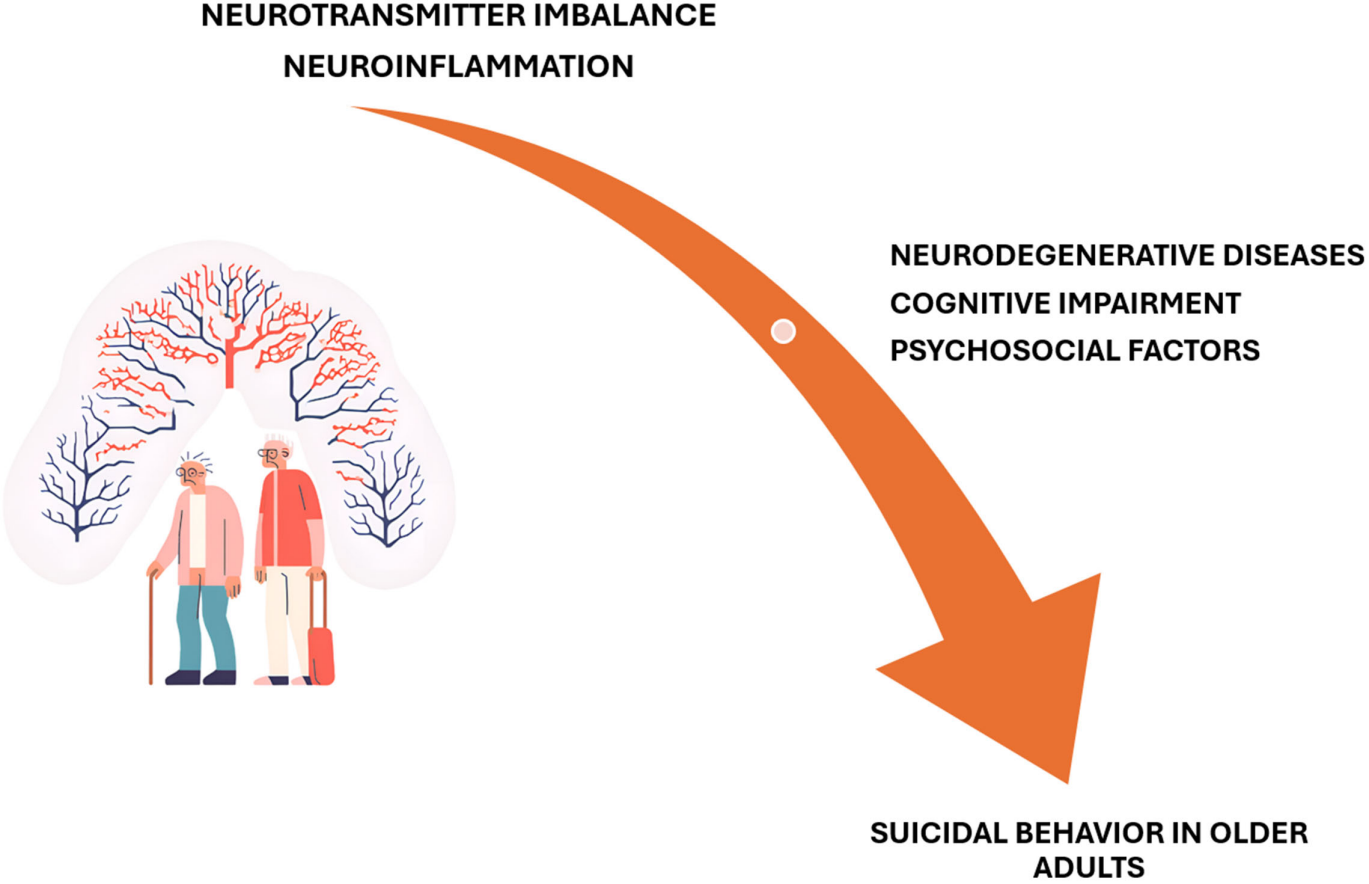
The neurotransmitter imbalance and neuroinflammation represent a substrate for suicidal behavior in older adults.

As summarized in **Figure 2**, the factors that lead to suicidal behavior in older adults are multifaceted and therefore the approach to dealing with such patients would also be complex. Education, improving social support, offering mental health care, and addressing financial concerns are critical aspects. Thus, learning these factors will help in coming up with a more fitting solution to the problem of suicide and enhance the quality of life of older adults.

## 4 Medico-legal aspects surrounding suicides in older adults

The medico-legal issues of suicides in older adults is a particular field of interest. A recent Italian retrospective study demonstrated that a great number of suicides occurs in men, with a higher rate after 80 y.o (113). Suicidal ideation and behavior can sometimes be difficult to identify and appropriately intervene due to the presence of co-morbid conditions, cognitive changes, and communication difficulties in older adults (7, 114). Additionally, assessing the capacity of older adults to make decisions regarding their care and treatment, including the decision to end their lives, raises intricate ethical and legal considerations (115, 116). Another crucial aspect concerns forensic investigations in cases of death at home of older adults: In the light of post-mortem events, this occurrence, from a forensic perspective, might be a characteristically challenging event. Furthermore, the concern for end-of-life considerations such as promoting palliative and hospice care, advanced directives, and ethical dilemmas concerning suicides and older adults further overlap with the medico-legal imbalance of the focus on older adult suicide cases, which requires a highly sensitive and systematic approach to address such concerns.

### 4.1 Forensic investigations

Forensic investigations in cases of suicide among older adults involve meticulous procedures to establish the cause of death accurately, differentiate between natural, accidental, and intentional causes, and rule out foul play (117, 118). These procedures encompass several key aspects: crime scene investigation, determination of postmortem interval (PMI), identification of the cause of death, autopsy challenges, toxicological examination, and histological tests (119). In similar cases, the victim lived alone, and their body was found with a long PMI present significant challenge due to severe post-mortem modifications (120). These cases often involve advanced decomposition, which complicates the determination of the cause and manner of death: given the long PMI and in relation of the external factor, such as the seasonality, the body may be in an advanced state of decomposition, mummification, or skeletonization (121). Investigators look for contextual evidence such as suicide notes, medications, and the arrangement of the living space to gather clues about the victim’s intentions and mental state. Environmental factors such as temperature and humidity, which affect the decomposition rate, are also recorded. Moreover, in such cases, because of climate change, post-mortem modifications could be accelerated (122). In similar cases, a multidisciplinary approach is essential, combining detailed crime scene analysis, entomology, pathology, and toxicology to establish the cause and manner of death.

A crucial aspect is the so-called psychological autopsy that involves reconstructing the psychological state of the deceased through interviews with family members, caregivers, and healthcare professionals (123). In geriatric suicides, the psychological autopsy plays a crucial role in elucidating the individual’s mental health history, recent life events, and behavioral changes that may have preceded the suicide (124). All these pieces of information could be very important not only in order to define the cause of death, but also to adopt preventive strategies and interventions. Identifying the precise cause of death in older adult suicides involves distinguishing between lethal injuries and underlying natural diseases. Common methods of suicide in older adults include overdosing on medications, hanging, precipitation, and firearm use: each method presents different challenges (125, 126). For instance, distinguishing a medication overdose from natural death due to disease requires thorough toxicological analysis. In cases involving hanging or firearms, the forensic pathologist must differentiate between suicidal injuries and potential post-mortem artifacts or pre-existing conditions.

**Figure 3:**
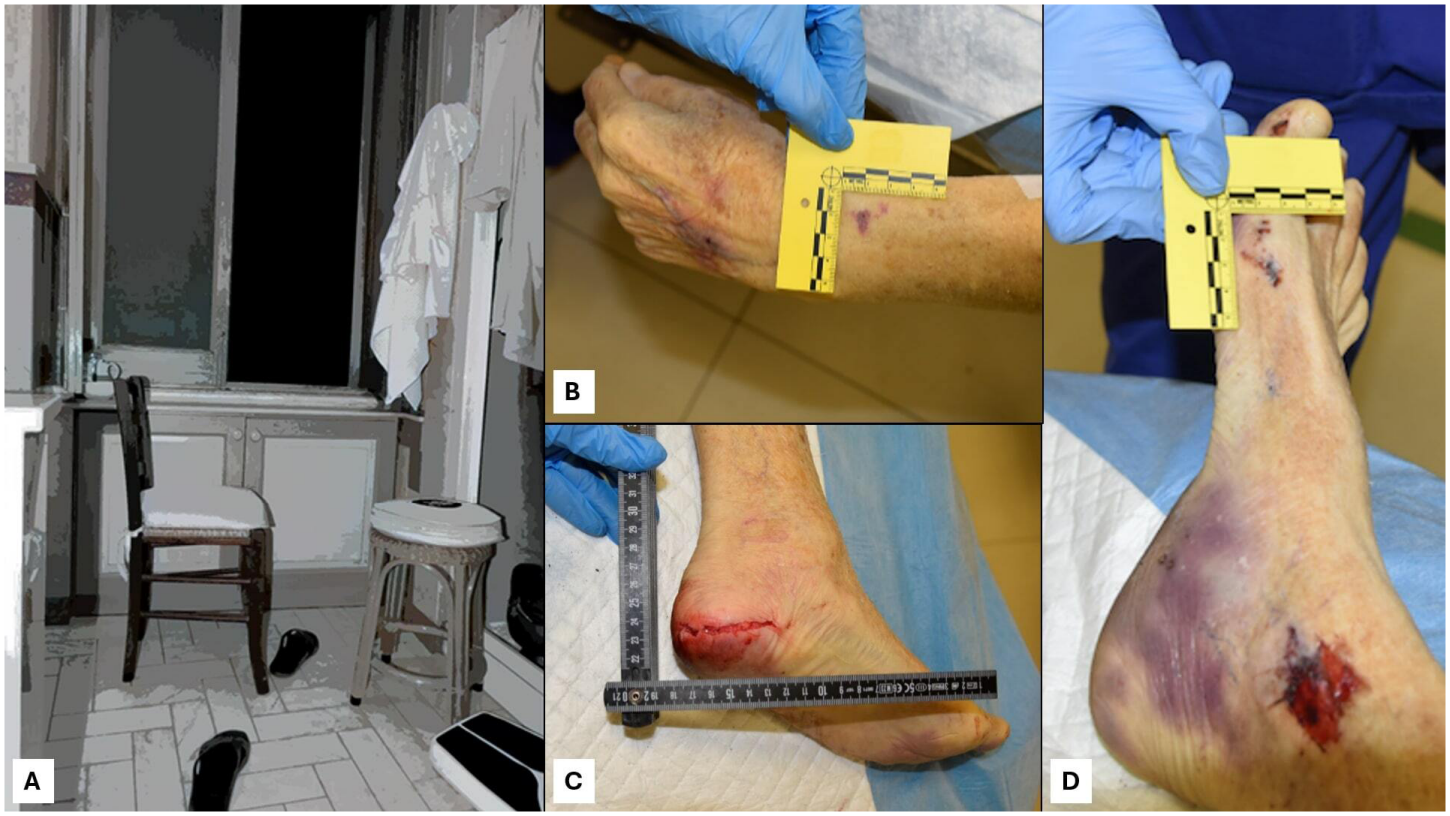
Crime scene examination in the case of suicide through precipitation **(A)**. At the external examination, each lesion should be carefully described and photographed **(B, C, D)**.

The initial phase of any forensic investigation begins at the crime scene. In cases of older adult suicide, investigators must proceed with sensitivity and precision: in this way the use of modern tools, such as the laser scanner technology, could be very useful (127, 128). The scene should be carefully documented through photographs, notes, and sketches to capture every detail. Investigators look for evidence such as suicide notes, medications, and the position of the body, which may provide clues about the method of suicide. The environment is examined for signs of a struggle or forced entry to rule out foul play (14). As represented in the case reported in **Figure 3**, the CSI investigation is fundamental in order to define the cause of death (**Figure 3A**). Moreover, the external examination of the corpse is very useful in describing the lesions, such as in the case of death through precipitation, where the lesions should be compatible with the dynamics of the fall (**Figures 3B-D**).

The inspection with a forensic light could be very useful to collect biological evidence: in this context, it is crucial to remember that collecting ‘touch DNA’ can be useful for placing a subject at a crime scene. However, this type of evidence should be handled with caution to accurately assess its significance in the context (129, 130). In older adult cases, special attention is given to potential contributory factors such as chronic illnesses, mobility aids, and living conditions, which might influence both the method and the likelihood of suicide (131).

Estimating the postmortem interval (PMI), or the time elapsed since death, is crucial in forensic investigations. This determination can be particularly challenging in older adults because of the presence of age-related physiological changes and potential comorbidities. Standard methods to estimate PMI include assessing body temperature, rigor mortis, and livor mortis. However, these methods can be influenced by the individual’s age and health condition. For instance, an older adult with decreased muscle mass may exhibit rigor mortis differently. Moreover, the use of new reliable methods to estimate the PMI, for example using the biochemistry of the vitreous humor, could be very useful (132). In the same manner, forensic entomology, which involves studying insect activity on the body, can also provide PMI estimates, however, it has to be interpreted carefully, considering the deceased’s environment and health (133). Furthermore, the application of histological and immunohistochemical investigations combined with standard methods could be fundamental in the correct estimation of the PMI and trauma dating (134, 135).

Conducting autopsies on older adults poses unique difficulties. Age-related diseases such as osteoporosis, arteriosclerosis, and organ atrophy can complicate the interpretation of findings. An autopsy must be meticulous, considering the delicate condition of older adult tissues. Pathologists must distinguish between injuries caused by suicide and those resulting from natural aging processes or pre-existing diseases. In addition, determining the impact of chronic illnesses on the cause of death is essential, as these conditions might mask or mimic trauma (136, 137).

During the autopsy, the forensic pathologist examines the body for evidence of self-inflicted injuries, such as ligature marks, gunshot wounds, or drug overdose (138, 139). Determining the presence and nature of these injuries is critical for confirming the cause of death and differentiating suicides from accidental deaths or homicides. It is important to acquire the medical conditions of the involved subject, including chronic illnesses, cognitive impairments, and physical frailty, which can complicate the forensic investigation of suicides (140, 141). The presence of comorbidities and the use of multiple medications require careful consideration during autopsy and toxicological analysis to ascertain the role of these factors in the individual’s mental state and decision-making processes. Toxicological analysis is critical in cases where drug overdose is suspected. Indeed, the analysis of biological samples, such as blood and urine, is essential for identifying the presence of drugs, alcohol, or other substances that may have influenced the individual’s behavior (142, 143). In geriatric suicides, the interaction between prescribed medications and their potential contribution to suicidal behavior necessitates a comprehensive toxicological assessment (144). Given the high prevalence of prescription medication use among older adults, toxicologists must identify and quantify multiple substances. Interpreting toxicology results can be complex because of polypharmacy (the use of multiple medications), which is common in this age group (145, 146). The presence of therapeutic, subtherapeutic, or toxic levels of medications must be carefully analyzed to ascertain whether the overdose was intentional. Additionally, interactions between medications, as well as the genetic substrate, can complicate the determination of toxicity levels (147).

Histological examinations involve microscopic analysis of tissues and are vital for identifying pathological changes at the cellular level. In older adults, histological tests help distinguish between age-related tissue changes and pathological findings relevant to the cause of death (148, 149). Moreover, the use of these techniques is fundamental to investigate vitality markers (150). For instance, chronic inflammation, fibrosis, or neoplastic changes must be evaluated in the context of potential trauma or poisoning. Histology can also reveal subtle signs of disease processes such as myocardial infarction or cerebral hemorrhage, which may contribute to the overall understanding of the cause of death (151). Moreover, as recently described in a thematic review, the post-mortem investigation on brain tissue could be very useful to better define the role of neurotrophin factors in suicide. In the same review, a pivotal role seems to be played by brain-derived neurotrophic factor (BDNF), while less evidence supports the hypothesis of glial cell line derived neurotrophic factor (GDNF) involvement (152). Furthermore, studies have shown that decreased gray matter volume in the hippocampus is associated with impaired memory and emotional dysregulation, which are factors in suicidal behavior. Integrity of white matter tracts, crucial for communication between brain regions, can be assessed using diffusion tensor imaging (DTI). Changes in white matter integrity may indicate disruptions in neural connectivity associated with mental disorders (153).

### 4.2 Legal framework and policy considerations in older adult suicide

The phenomenon of suicide among older adults poses significant ethical, legal, and policy challenges (154). As society grapples with the complexities of an aging population, it is crucial to develop and implement a robust legal framework and comprehensive policy measures to address this sensitive issue. These frameworks mustrespect individual autonomy while protecting vulnerable populations, ensuring access to mental health care, and promoting preventive strategies (155).

Considering the legal framework, one of the most contentious legal issues related to older adult suicide is the question of older adult abuse and neglect, which can be contributing factors to suicidal ideation among older adults (6, 156). The types of older adult abuse could be:

- physical abuse: inflicting physical pain or injury through actions such as hitting, pushing, or inappropriate use of restraints;
- emotional abuse: causing emotional pain or distress through verbal assaults, threats, humiliation, or harassment;
- sexual abuse: non-consensual sexual contact of any kind with an older adult;
- financial abuse: illegal or unauthorized use of an older adult’s funds, property, or assets;
- neglect: failure to provide the necessary care, including food, shelter, healthcare, and protection.

Several factors contribute to older adult abuse, including caregiver stress, societal attitudes that devalue the older adult, and their isolation or dependence on others for care. Cognitive impairments such as dementia also increase vulnerability (157, 158). Prevention strategies may be the improvement of caregiver and healthcare professional education, particularly in the identification of signs of older adult abuse and the importance of reporting them. As recently reported, home care might positively impact on preventing suicidal behavior in individuals with dementia, however, further investigation is needed (159). Moreover, it is essential to provide resources and support for caregivers to reduce stress and prevent burnout (160). Furthermore, public health campaigns could be useful to identify the signs of depression and suicidal ideation in older adults, helping family members, caregivers, and healthcare providers to intervene early (161). Another crucial aspect concerns social isolation, which represents a significant risk factor for suicide, and initiatives to promote social engagement and community involvement can mitigate this phenomenon (99). Programs that encourage intergenerational activities, volunteerism, and community-based services can help older adults stay connected and reduce feelings of loneliness and worthlessness. Finally, the legal validity of advanced directives and the determination of decision-making capacity in the context of suicidal behavior pose important medico-legal challenges that require careful consideration within the legal and healthcare systems: strengthening laws and regulations, as well as establishing community programs, may protect individuals and reduce risks (162).

### 4.3 Ethical considerations in older adult suicide

It is therefore very clear that because of the complex nature of legal and ethical issues, the prevention and combating of older adult abuse and neglect involves a delicate mix of legal and ethical approaches (163). Legal systems give the necessary means to safeguard older adults by means of the reporting requirements, use of protective services, criminal sanctions, and guardianship provisions (164). However, these measures have to be carried out following a firm ethical framework that recognizes self-determination, privacy, and the worth of older adults (165). Through enforcing appropriate and strong legal frameworks and combining them with ethical issues concerning older adults, society will be in a good position to protect older adults by giving them the dignity they deserve.

One of the hardest questions in ethical decision-making in the case of older adult abuse is the conflict of respect for the autonomy of older adults and the need to protect them. Older adults have the same rights as everybody else, including the right to self-determination – that means the right to stay alone or reject some forms of treatment (166). However, when there is a threat to their lives, it becomes necessary to intervene and take them away from such situations. As much as people must be allowed to decide about their own body, especially in the case of patients with mental disorders, it becomes a Herculean task for healthcare and legal practitioners. That is why ethical practice entails making sure that the particular intervention is the least intrusive and as much as possible, in accordance with the patient’s wishes and their rights as a person (167).

The major principles of beneficence, the principle of doing good, and nonmaleficence, the principle of doing no harm, apply to elder care. This means that caregivers and professionals have a duty of implementing the best interest of older adults and make sure the action taken will not be detrimental to their welfare (168). This includes meeting the needs of older adults through caregiving, fighting for their rights, and ensuring that they are shielded from abuse or mistreatment.

It implies that all older adults should be treated right and accorded equal rights and privileges irrespective of their economic status, color or origin (169). Ethical practice demands that all older adults have to be provided with protection and care, and for this to be made possible, resources, support, and interventions should be distributed (115).

Furthermore, issues related to end-of-life care, decision making and the controversy regarding the permissibility of palliative and hospice care for older adult suicides are also included in the medico-legal domain (116). The challenge of intervening in the lives of older adults who may be vulnerable to self-neglect is a contentious one, thus there is a fine line between honoring their right to independence and doing what is best for them. Undoubtedly, each case should be treated as unique, it is necessary to assess each aspect in order to identify the nature of the event.

### 4.4 Mental health assessments in post-mortem samples in older adult suicide cases

Psychological evaluations of the brain in the post-mortem specimens are important for identifying the antecedent causes and precipitating factors in older adult suicides (170). These assessments entail evaluating aspects of biology, psychology, and the social environment that may have prompted the individual to suicide (171). Forensic samples taken after death can help identify any mental health issues and stress factors that the older adult was suffering from, which can further enhance the understanding and may even suggest measures to avoid such incidents in the future (172).

The above discussion shows that one of the primary objectives of post-mortem mental health assessment is a neurochemical examination. This includes analyzing mood regulating neurotransmitters such as serotonin, DA, and NE among others that are related to mental health conditions. Low levels of certain neurotransmitters, such as serotonin, have been linked to depression and suicidal tendencies (173). When these neurochemical levels are measured in brain tissue samples, it may be possible to determine the mental state of the person.

The correlations between chronic stress and mental health disorders are associated with changes in stress biomarkers such as cortisol (174). Cortisol is a hormone that is released during stress and since stressed people have high cortisol levels, it is possible to get this hormone even from hair of a dead body. These biomarkers can work to substantiate a history of chronic stress or anxiety, both of which are suicide risk factors (175). New data suggest that oxidative stress may be implicated in suicidality, but only one study has examined the sources of reactive oxygen species (ROS) in subjects demonstrating suicidal behavior. It has been demonstrated that oxidative stress by nicotinamide adenine dinucleotide phosphate (NADPH) oxidase (NOX2) is involved in the changes in the animal model of psychosis. According to these studies, the raised NOX2 mediated oxidative stress in the brain might participate in the neuropathological alterations responsible for suicidal behavior (176, 177).

In addition, histological analysis of brain samples can show the histopathological abnormalities that are related to mental illnesses. For example, the volume of the hippocampus was shown to be decreased in patients with major depressive disorder. By analyzing samples of brain tissue, pathologists can discover such morphological alterations that may have given rise to the psychological state of the patient (178).

Molecular and genetic analyses can help to determine the presence of genetic and epigenetic factors that may affect the individual’s psychological state. In human samples, we can study the depressed or activated genes in the brain to determine whether they are linked to depression, anxiety, or other mental disorders (179, 180). As recently demonstrated, major depressive disorder could be related to miRNA dysregulation both in peripherical blood and in post-mortem brain samples (181, 182). It can also assist in understanding the various biological factors that could have contributed to an older adult’s suicide. In addition, as highlighted in the previous section, the toxicological data play an important role in post-mortem evaluation of mental health issues, especially in older adult suicide. In direct post-mortem assessment, there is the concentration of the biological and chemical results, but there is the consideration of the medical and personal background of the deceased. Data from medical records such as patient’s history of mental illnesses, previous suicide attempts, and prescribed medications can provide crucial background information (183).

Therefore, it is imperative that an extensive psychiatric evaluation is conducted to properly assess the risk of suicide in older adults. Capacity assessment is not easy for forensic investigators to handle because of the numerous aspects of an older adult’s suicide they must work within.

Furthermore, histological analysis of the brain tissues also depicts some structural alterations that are related to mental health disorders. For instance, it was revealed that the hippocampal volume is reduced in major depressive disorder. The pathologists can then tell such things from the brain tissue samples that might have caused such structural changes that probably caused the individual’s psychological problems.

## 5 Discussion

Older adults often suffer from neurological disorders such as Alzheimer’s, Parkinson’s, and vascular dementia, leading to significant mental health changes, impaired performance, and decision-making, which can result in suicidal tendencies (184, 185). Neuropsychological assessments are crucial for detecting cognitive changes and potential confounding factors, helping clinicians understand the link between brain function, mental health, and suicide risk (186).

Neurochemical markers, such as neurotransmitters and their metabolites, can provide insights into the biochemical environment of the brain before death. These markers are often studied post-mortem and can indicate abnormalities or dysregulations that may have contributed to the individual’s mental state and behavior. Neurotransmitters, such as serotonin, DA, and NE, play crucial roles in regulating mood, cognition, and behavior. Post-mortem studies examine levels of these neurotransmitters and their metabolites in brain tissues (35, 187). In the same way, inflammatory markers, including cytokines and microglial activation, are increasingly recognized for their role in neuropsychiatric disorders (21, 47). Moreover, structural changes in the brain, observed through neuroimaging or post-mortem analysis, provide anatomical insights into how these changes may correlate with mental health conditions and behaviors exhibited during life. Reduction in gray matter volume, particularly in regions associated with mood regulation such as the prefrontal cortex and hippocampus, are common in various mental disorders (153). Combining neurochemical analyses with structural brain changes provides a more comprehensive understanding of the neurobiology underlying mental states before death. In addition, identifying specific neurochemical markers or structural brain changes associated with suicidal behavior can inform diagnostic criteria, treatment strategies, and preventive measures. Neurochemical markers in tissues and structural brain changes observed post-mortem offer valuable insights into the pre-mortal mental state. These markers can illuminate underlying neurobiological mechanisms that contribute to mental disorders and suicidal behavior, paving the way for targeted interventions and improved patient care strategies.

Forensic evaluation in older adults is very useful to gather information for the formulation of preventive measures to tackle the risks and weaknesses in this vulnerable group of the population. By comparing the identified precipitating factors and patterns, the forensic findings can be useful in providing the necessary guidance for the provision of specific interventions, mental health support services and social policies that would help prevent suicide in older adults (4, 188). Furthermore, because formalin-fixed, paraffin-embedded tissue analysis makes it feasible, post-mortem samples obtained for other uses—such as histological examination to determine organ damage—may be used. Thus, forensic research facilities serve as a priceless “magic box” for obtaining samples, underscoring the significance of upholding moral principles such as the Declaration of Helsinki.

Patients who are older adults and present with suicidal thoughts in the emergency department need to be assessed differently. Practical considerations, such as living in the countryside, availability of mental health care, and legal issues (forcible admission, for instance) should also be considered. Psychiatric and psychological interventions are also ideal for older adults and should be offered in a comprehensive manner. This includes management of mental health disorders (for instance depression, bipolar disorder, anxiety) which are associated with a high suicide rate among this group of people (189, 190).

These are comprehensive in the sense that they involve medical, psychological, and social interventions in dealing with suicidal behaviors among older adults (7). One of these is to improve mental health care for older adults, such as increasing the number of geriatric psychiatrists, counselors, and support group meetings (102). Raising awareness on potential suicidal indicators in older adult patients and encouraging healthcare practitioners, caregivers, and family members to discuss mental health and end-of-life preferences are crucial for timely identification and prevention (102). Additionally, practices of screening for depression, anxiety and the relative genetic predisposition investigation, cognitive changes during routine health check-ups can also assist in early detection of these patients and early management. Social disconnectedness and loneliness are critical predictors of geriatric suicides; therefore, social, community-based organizations, senior citizen centers, and support groups are crucial in reducing feelings of isolation amongst seniors (191). Further, older adults who experience financial difficulties, who require help with basic physical needs, and those who cannot afford medical or mental healthcare services are vulnerable to suicidal thoughts. Through these proactive measures, society can establish the necessary framework that could protect the mental health of older adults and decrease the rate of geriatric suicides.

Based on these considerations, community intervention programs offer important assistance and recommendations for older adults who are prone to suicide (192, 193). Such programs may be a joint effort of the health care centers, social welfare offices, and other community-based organizations wherein they provide different services such as screening to detect cases of mental illnesses, counseling to individuals and families, and setting up seminars and/or lectures on the topic of suicide prevention. Furthermore, they could help to arrange social activities, volunteering, and peer-support groups, which are important to prevent social isolation and loneliness that are risk factors for geriatric suicide. In addition, community outreach programs can also help in building relationships between older adults and younger people, where older adults can volunteer to mentor younger people or take up community service, thus giving them a sense of direction and usefulness. Therefore, community outreach programs remain an effective means of fostering optimal health, as well as reducing geriatric suicide risk, by increasing social acceptance and belonging for older adults (194).

## 6 Conclusion

Older adult suicide represents a significant and complex challenge, intricately linked to neurotransmitter imbalances and underscored by crucial medico-legal considerations. Neurotransmitters are known to influence mood regulation and can significantly contribute to suicidal ideation in older adults. Given that mental health disorders such as depression are prevalent among older adults, addressing these imbalances is critical for developing targeted interventions. A thorough understanding of the complex interactions between neurotransmitter imbalances and mental health is essential for accurate medico-legal evaluations of suicide. Such evaluations must consider not only the biological but also the psychological and social dimensions of older adult suicides. This interdisciplinary approach can lead to identifying strategies for the identification, prevention, and ethical management of suicidal behavior in older adults. The need for specialized measures to screen for suicidal ideation in this age group is evident, as conventional assessment tools may not fully capture the unique neurobiological and psychosocial factors affecting older adults. Specialized instruments designed for older adults should incorporate factors such as age-related cognitive decline, chronic illness, and social isolation, which are often overlooked in non-specific measures. Ongoing research must promote closer collaboration among neurobiologists, clinical practitioners, and legal professionals to deepen our understanding of the neurobiological mechanisms behind suicidal behavior and develop more effective prevention strategies.
